# Is photobiomodulation an effective preventive strategy for oral mucositis in patients with hematologic diseases undergoing chemotherapy?: A systematic review of randomized controlled trials

**DOI:** 10.1007/s10103-026-04814-7

**Published:** 2026-02-28

**Authors:** Maria Gabriella Apolinário Xavier, Alícia Marcelly Souza de Mendonça Silva, Guilherme Rodrigues Wanderley de Oliveira, Allan Vinícius Martins-de-Barros, Stefânia Jeronimo Ferreira, Raylane Farias de Albuquerque, Igor Henrique Morais Silva, Lucas Nascimento Ribeiro

**Affiliations:** 1 Residents in Hospital Dentistry with a Focus on Oncology at the Pernambuco Cancer Hospital, Recife, Brazil; 2 University of Pernambuco, School of Dentistry of Arcoverde, Arcoverde, Pernambuco Brazil; 3 Department of Dentistry at the Pernambuco Cancer Hospital, Recife, Brazil

**Keywords:** Photobiomodulation, Oral mucositis, Chemotherapy, Hematologic diseases

## Abstract

Purpose to conduct a systematic review to assess whether photobiomodulation is effective in preventing oral mucositis in patients with hematologic diseases undergoing chemotherapy. Methods: A systematic search was conducted in the Embase, Medline/PubMed, Cochrane, Scopus, Web of Science and LILACS databases. The strategy included selected randomized clinical trials that evaluated the prevention of oral mucositis, directly comparing the use of photobiomodulation for chemotherapy-induced oral mucositis in patients with hematologic diseases with alternative non-laser therapies. The risk of bias in the studies was assessed using the Cochrane Collaboration’s tool for assessing risk of bias and the certainty of evidence was assessed using GRADE. Results: After applying all predetermined criteria, six randomized clinical trials, dated between 1997 and 2024, were included. A total of 200 patients were evaluated, with all six studies using the red wavelength, with only one study using a combination with the infrared wavelength. It was observed that photobiomodulation can be effective in preventing oral mucositis, reducing its frequency. Furthermore, a reduction in pain intensity and an improvement in quality of life were also identified. Conclusion: The use of photobiomodulation shows promising results in preventing oral mucositis, consequently reducing the frequency and pain of patients with hematologic diseases undergoing chemotherapy.

## Introduction

According to the Global Cancer Observatory (Globocan) estimates, approximately 2.10 million cases of onco-hematological disorders are expected worldwide by 2045. Among the most frequent are: Non-Hodgkin’s Lymphoma, Leukemias, Hodgkin’s Lymphoma and Multiple Myeloma. Factors such as Human Immunodeficiency Virus (HIV), genetics, exposure to chemicals and ionizing radiation are closely related to the incidence of such diseases [1].

Regarding approaches to treatment methods, chemotherapy (CT) protocols associated or not with Hematopoietic Stem Cell Transplantation (HSCT) are defined as the gold standard for patients diagnosed with hematologic malignancies. Furthermore, bone marrow transplantation is also considered for the management of non-malignant hematologic diseases, such as severe aplastic anemia, myelodysplastic syndromes and sickle cell anemia [2–4].

Despite the curative effects and improved patient survival, a series of acute or late toxicities caused by certain CT drugs can result in treatment interruption, increased risk of secondary infections and, consequently, longer hospital stays. In the oral cavity, cytotoxic effects are perceived in mucous membranes and salivary glands, influencing the morbidity of individuals [5–7]. Fungal infections and oral mucositis (OM) are the most common oral manifestations observed in these patients [8], in addition to xerostomia, vascular disorders, trismus and dentoalveolar abscess [9, 10].

OM is identified as an inflammation that can affect the entire oral cavity with erythema and/or ulcerations of varying extent, in addition to directly interfering with the patient’s Quality Of Life (QOL) and systemic conditions [11, 12]. Photobiomodulation (PBM) has been widely used as an alternative in the prevention and treatment of chemoinduced oral mucositis, due to its cost-effectiveness and biological effects, such as reduced tissue damage and improved pain perception levels [13, 14]. However, dosimetric aspects and their correlation with chemotherapy protocols have not yet been fully understood [15–17]. Given the above, this review seeks to systematically evaluate the preventive efficacy of PBM in OM in patients with hematologic diseases undergoing CT.

Despite the growing body of evidence on the role of PBM in the prevention and management of OM, a significant gap persists in the literature regarding the standardization of dosimetric parameters used and the heterogeneity of CT protocols among studies. Although several studies demonstrate the clinical benefits of PBM, few rigorously synthesize, considering exclusively randomized clinical trials, which dose, energy, wavelength, and application frequency configurations present the greatest preventive efficacy in patients with hematological diseases undergoing CT. Furthermore, it is still unclear whether the therapeutic response to PBM varies according to different CT regimens. Thus, this systematic review seeks to fill this gap by comprehensively and comparatively evaluating the preventive effectiveness of PBM in OM, focusing on methodologically robust studies, allowing for greater clarity and scientific support for the definition of standardized clinical protocols.

## Materials and methods

### Registration and protocol

The methods of this systematic review were registered in The International Prospective Register of Systematic Reviews (CRD420251053168) and its entire conduction followed the PRISMA checklist (Preferred Reporting Items for Systematic Reviews and Meta-analyses) [18].

### Eligibility criteria

Eligibility criteria were based on the PICO question: “Is photobiomodulation effective in preventing oral mucositis in patients with hematologic diseases undergoing chemotherapy?“. Population: Patients with hematologic diseases undergoing chemotherapy; Intervention: Photobiomodulation; Control: Placebo or simulated PBM or alternative therapies without laser application; Primary outcome: Prevention of OM; Secondary outcomes: Effectiveness of OM treatment after failure of preventive intervention, pain and QOL.

Randomized clinical trials (RCTs) that prospectively follow and evaluate patients with hematologic diseases, directly comparing the use of PBM with alternative therapies or without the use of laser for the prevention of chemotherapy-induced OM were assessed for eligibility. To be considered eligible, studies should include data regarding the diagnosis of the hematologic diseases, the CT protocol the patient underwent, the dose of PBM, the wavelength, the method and frequency of OM assessment, and the type of classification system used to assess OM. Observational studies, case reports, case series, letters to the editor, and animal studies were excluded.

### Information sources and search strategy

A systematic literature search was conducted using the following databases: Embase, Medline/PubMed, Cochrane, Scopus, Web of Science and LILACS. The search strategy was developed in the PubMed platform and adapted for other databases, using the terms: (“Hematologic Disease” OR “Hematologic Neoplasms” OR Haematological OR “Haematological Malignancies” OR Leukemia OR Lymphoma) AND (“Laser Therapy” OR “Low-Level Light Therapy” OR LLLT OR Photobiomodulation OR “Photodynamic therapy”) AND (Mucositis OR “Oral Mucositis”). Synonyms of the terms Mesh were also included to broaden the literature search. No limitations regarding language, year, or type of publication were applied to the search. The strategies used for each database are listed in Table 1. A manual search was performed in the references of the included articles and in the main journals on the topic: Supportive Care in Cancer, Lasers in Medical Science, Medicina Oral Patologia Oral y Cirurgia Bucal and Oral diseases.

**Table 1:** Search strategy

Database	Search strategy	Filter
Embase	(‘hematologic disease’/exp OR ‘hematology’/exp OR ‘hematologic malignancy’/exp OR ‘lymphoma’/exp OR ‘leukemia’/exp) AND ‘oral mucositis’/exp AND (‘laser therapy’/exp OR ‘low level laser therapy’/exp)	No filters applied
PubMed/MEDLINE	(“Hematologic Neoplasms“[All Fields] OR (“haematological“[All Fields] OR “haematologically“[All Fields] OR “hematological“[All Fields] OR “hematologically“[All Fields] OR “hematology“[MeSH Terms] OR “hematology“[All Fields] OR “haematologic“[All Fields] OR “hematologic“[All Fields]) OR “Haematological Malignancies“[All Fields] OR (“leukaemia“[All Fields] OR “leukemia“[MeSH Terms] OR “leukemia“[All Fields] OR “leukaemias“[All Fields] OR “leukemias“[All Fields] OR “leukemia s“[All Fields]) OR (“lymphoma“[MeSH Terms] OR “lymphoma“[All Fields] OR “lymphomas“[All Fields] OR “lymphoma s“[All Fields])) AND (“Laser Therapy“[All Fields] OR “Low-Level Light Therapy“[All Fields] OR (“Low-Level Light Therapy“[MeSH Terms] OR (“low level“[All Fields] AND “light“[All Fields] AND “therapy“[All Fields]) OR “Low-Level Light Therapy“[All Fields] OR “lllt“[All Fields]) OR (“Low-Level Light Therapy“[MeSH Terms] OR (“low level“[All Fields] AND “light“[All Fields] AND “therapy“[All Fields]) OR “Low-Level Light Therapy“[All Fields] OR “photobiomodulation“[All Fields]) OR “Photodynamic therapy“[All Fields]) AND (“mucosalization“[All Fields] OR “mucosalized“[All Fields] OR “mucosally“[All Fields] OR “mucose“[All Fields] OR “mucoses“[All Fields] OR “mucositis“[MeSH Terms] OR “mucositis“[All Fields] OR “mucositides“[All Fields] OR “mucous membrane“[MeSH Terms] OR (“mucous“[All Fields] AND “membrane“[All Fields]) OR “mucous membrane“[All Fields] OR “mucosal“[All Fields] OR “Oral Mucositis“[All Fields])	No filters applied
Clinicaltrials.gov	(“Hematologic Neoplasms” OR Haematological OR “Haematological Malignancies” OR Leukemia OR Lymphoma) AND (Mucositis OR “Oral Mucositis”) AND (“Laser Therapy” OR “Low-Level Light Therapy” OR LLLT OR Photobiomodulation OR “Photodynamic therapy”)	No filters applied
LILACS	(“Hematologic Neoplasms” OR Haematological OR “Haematological Malignancies” OR Leukemia OR Lymphoma) AND (“Laser Therapy” OR “Low-Level Light Therapy"OR LLLT OR Photobiomodulation OR “Photodynamic therapy”) AND (Mucositis OR “Oral Mucositis”)	No filters applied
Scopus	(“Hematologic Neoplasms” OR haematological OR “Haematological Malignancies” OR leukemia OR lymphoma) AND (“Laser Therapy” OR “Low-Level Light Therapy” OR lllt OR photobiomodulation OR “Photodynamic therapy”) AND (mucositis OR “Oral Mucositis”)	No filters applied
Web of Science	(“Hematologic Neoplasms” OR Haematological OR “Haematological Malignancies” OR Leukemia OR Lymphoma) AND (Mucositis OR “Oral Mucositis”) AND (“Laser Therapy” OR “Low-Level Light Therapy” OR LLLT OR Photobiomodulation OR “Photodynamic therapy”)	No filters applied
Cochrane	“Hematologic Neoplasms” OR Haematological OR “Haematological Malignancies” OR Leukemia OR Lymphoma AND “Laser Therapy” OR “Low-Level Light Therapy” OR LLLT OR Photobiomodulation OR “Photodynamic therapy” AND Mucositis OR “Oral Mucositis” (Word variations have been searched)	No filters applied
Supportive Care in Cancer	“Hematologic Neoplasms” OR Haematological OR “Haematological Malignancies” OR Leukemia OR Lymphoma AND “Laser Therapy” OR “Low-Level Light Therapy” OR LLLT OR Photobiomodulation OR “Photodynamic therapy” AND Mucositis OR “Oral Mucositis” (Word variations have been searched)	Research article
Lasers in Medical Science	“Hematologic Neoplasms” OR Haematological OR “Haematological Malignancies” OR Leukemia OR Lymphoma AND “Laser Therapy” OR “Low-Level Light Therapy” OR LLLT OR Photobiomodulation OR “Photodynamic therapy” AND Mucositis OR “Oral Mucositis” (Word variations have been searched)	Research article
Medicina Oral, Patologia Oral y Cirurgia Bucal	“Hematologic Neoplasms” OR Haematological OR “Haematological Malignancies” OR Leukemia OR Lymphoma AND “Laser Therapy” OR “Low-Level Light Therapy” OR LLLT OR Photobiomodulation OR “Photodynamic therapy” AND Mucositis OR “Oral Mucositis” (Word variations have been searched)	No filters applied
Oral diseases	“Hematologic Neoplasms” OR Haematological OR “Haematological Malignancies” OR Leukemia OR Lymphoma AND “Laser Therapy” OR “Low-Level Light Therapy” OR LLLT OR Photobiomodulation OR “Photodynamic therapy” AND Mucositis OR “Oral Mucositis” (Word variations have been searched)	No filters applied
Current Controlled Trials	“Hematologic Neoplasms” OR Haematological OR “Haematological Malignancies” OR Leukemia OR Lymphoma AND “Laser Therapy” OR “Low-Level Light Therapy” OR LLLT OR Photobiomodulation OR “Photodynamic therapy” AND Mucositis OR “Oral Mucositis” (Word variations have been searched)	No filters applied
International Clinical Trials Registry Platform	“Hematologic Neoplasms” OR Haematological OR “Haematological Malignancies” OR Leukemia OR Lymphoma AND “Laser Therapy” OR “Low-Level Light Therapy” OR LLLT OR Photobiomodulation OR “Photodynamic therapy” AND Mucositis OR “Oral Mucositis” (Word variations have been searched)	No filters applied
ReBEC	“Hematologic Neoplasms” OR Haematological OR “Haematological Malignancies” OR Leukemia OR Lymphoma AND “Laser Therapy” OR “Low-Level Light Therapy” OR LLLT OR Photobiomodulation OR “Photodynamic therapy” AND Mucositis OR “Oral Mucositis” (Word variations have been searched)	No filters applied
EU Clinical Trials Registry	“Hematologic Neoplasms” OR Haematological OR “Haematological Malignancies” OR Leukemia OR Lymphoma AND “Laser Therapy” OR “Low-Level Light Therapy” OR LLLT OR Photobiomodulation OR “Photodynamic therapy” AND Mucositis OR “Oral Mucositis” (Word variations have been searched)	No filters applied

Two authors working independently (LNR and MGAX) reviewed the abstract and title of the search results. After searching each database, duplicate studies were removed using Rayyan software [19]. All potentially relevant articles were investigated by reading the full text. If there was a difference of opinion, consensus was reached by consulting a third author (RFA). To assess inter-rater agreement for study inclusion, the Kappa score was used. The scores obtained were analyzed as 0 (no agreement), < 0.8 (moderate agreement), or ≥ 0.8 (almost perfect agreement) [20].

To locate unpublished and ongoing trials related to the review question, clinical trial registries were also searched: Current Controlled Trials (www.controlled-trials.com), the International Clinical Trials Registry Platform (http://apps.who.int/trialsearch/), ClinicalTrials.gov (www.clinicaltrials.gov), Rebec (www.rebec.gov.br), and the EU Clinical Trials Register (https://www.clinicaltrialsregister.eu).

### Data extraction and collection process

Data extraction was performed by two independent evaluators (LNR and MGAX). Differences in results were discussed and resolved by mutual agreement between the evaluators. The following data were collected: study title, study author, year of publication, study design, participant age and sex, chemotherapy dose and type, laser/control application time, criteria and classification system for mucositis assessment, laser type, spot size (cm²), application points, energy per point (J), energy per session (J), dose (J/cm²), wavelength (nm), application mode, exposure time, application frequency, and study outcome and conclusion. In case of missing data, the corresponding author were contacted to retrieve this information.

### Evaluation of outcomes

The primary outcome of this review was to evaluate the prevention of OM in patients with hematologic diseases undergoing CT and exposed to PBM. We considered studies that evaluated patients with hematologic diseases undergoing CT that induce OM. Furthermore, we included studies that used instruments to measure OM symptoms using specific severity scales, and oral health-related QOL, focusing on dimensions such as pain, discomfort, oral function, and psychological impact. The secondary outcome was the efficacy of OM treatment after failure of preventive intervention, with emphasis on the evaluation of parameters such as pain intensity, QOL and oral mucosa healing during PBM treatment.

### Risk of bias within individual studies

Quality assessments of the selected studies were performed by three independent reviewers (LNR, MGAX, and RFA) using the Cochrane Collaboration’s tool for assessing risk of bias (RoB) in RCTs [21]. The assessment instrument contains six items: sequence generation, allocation concealment, blinding of outcome assessors, incomplete outcome data, selective outcome reporting, and other potential sources of bias. Each domain was judged as having low, high, or unclear bias. Each study was classified as having a low RoB (if all key domains were judged to have a low RoB), an unclear risk (if one or more key domains were judged to have an unclear risk), or a high RoB (if at least one key domain was judged to have a high RoB). When a study was classified as unclear, its authors were contacted to obtain further information to allow a definitive judgment.

### Certainty of evidence

The quality of evidence was assessed using the “Grading of Recommendations Assessment, Development and Evaluation” (GRADE) system, as recommended in the Cochrane Handbook for Developing Systematic Intervention Reviews version 5.1.0 [22]. This tool makes it possible to assess the quality of each result according to a 4-level classification: very low, low, moderate and high. The initial evidence quality rating is generated according to the study design (RCTs or observational study) and may be reduced according to methodological limitations (risk of bias), inconsistency, indirect evidence, imprecision, and publication bias, or it may be increased depending on the magnitude of the effect, dose-response gradient and residual confounders. The evaluation was carried out independently by two evaluators (LNR and MGAX) using the GRADEpro program. In cases of disagreement, a third reviewer (RFA) was consulted for resolution.

## Results

### Study selection

**Fig. 1:**
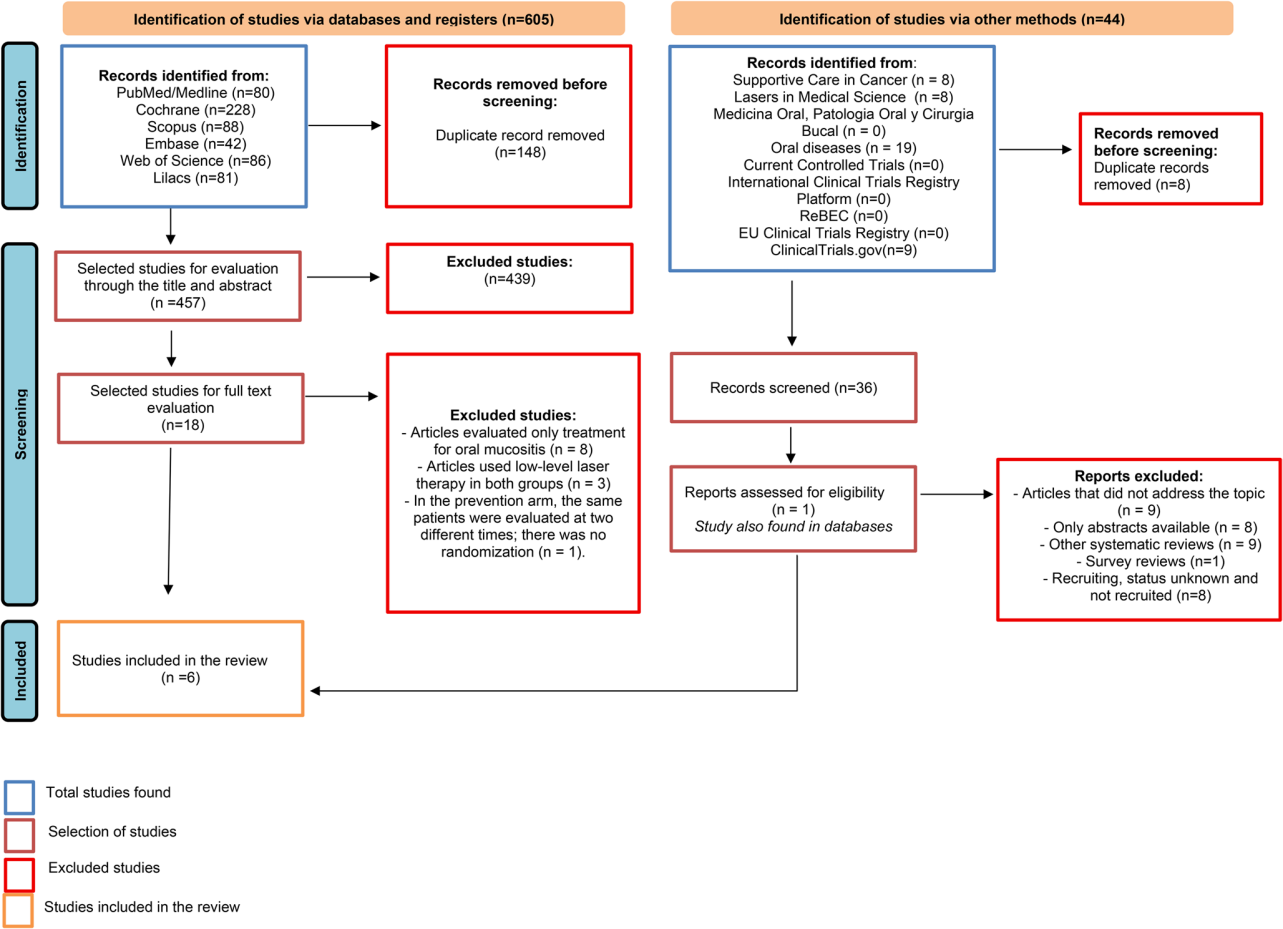
PRISMA 2020 flow diagram for new systematic reviews which included searches of databases, registers and other sources

A total of 605 studies were identified in this systematic review, selected from the Embase, Medline/PubMed, Cochrane, Scopus, Web of Science and LILACS databases. After removing duplicates, a total of 457 studies were screened and evaluated, of which 439 did not meet the pre-established eligibility criteria. Only 18 remaining studies passed to the full text evaluation stage, of which twelve were excluded due to methodological criteria and research design. After applying all the predetermined criteria, six RCTs [23–28] dated between 1997 and 2024 met the inclusion criteria and comprised the final analysis (Fig. 1). The agreement between the authors according to the Kappa test was 0.81, which means “almost perfect agreement”.

### Description of included studies

This systematic review included six RCTs that evaluated the efficacy of PBM as a preventive strategy for OM in patients with hematologic diseases undergoing CT. All studies randomly analyzed the effects of the laser therapy on intervention PBM and control groups (CG) (Simulated PBM and/or oral care). (Table 2).

**Table 2:** Summary of the characteristics of the studies

Study ID	Type of study	Age	Nº of subjects in intervention group/control group	Chemotherapy protocol in intervention group/control group	Type of hematologic disease in intervention group/control group	Intervention group	Control group	Time of laser/control aplication (prevention)	Time of laser/control aplication (treatment)	Criteria for evaluation of oral mucositis and others
Cowen D et al.., 1997 (France) [23]	A double blind randomized clinical trial	Mean age (38.4 years): (Range 18–58)	16 (9 F/7 M); 14 (6 F/8 M)	Cyclophosphamide (60 mg/kg - d5 and d4) + TBI (12 Gy - d3, d2 and d1): (14/13); Melphalan (140 mg/kg - d5) + TBI (Total of 12 Gy - d3, d2 and d1): (2/1).	Acute Lymphoblastic Leukemia (7/4); Acute Myeloblastic Leukemia (4/8); Non-Hodgkin’s Lymphoma (3/1); Multiple Myeloma (2/1); All received BMT (autologous).	PBM + Oral care	Simulated PBM	Applications daily for five consecutive days (from d-5 to d-1; before transplant)	Morphine was always given in case of grade III OM (Did not apply laser as therapeutic treatment).	Pre-established scale published [29] derived from Walsh et al. [30] and DMI and COMS.
Antunes HS et al., 2007 (Brazil) [24]	Randomized, placebo-controlled, quantity, and prospective clinical trial	Mean age: ≅ 37	19 (7 F/12 M); 19 (8 F/11 M)	*Regimen 1:* Cyclophosphamide 1800 mg/m2/d, d 6 and d 3; carmustine 450 mg/m2/d, d 2; etoposide 2400 mg/m2, d 7 (34 h). (5/5); *Regimen 2:* Cyclophosphamide 60 mg/kg/d, d 3 and d 2; TBI 22 Gy every 12 h, d 7 to d 5; antithymocyte globulin 15 mg/kg/d, d 5 to d 4. (3/5); *Regimen 3:* Cyclophosphamide 60 mg/kg/d, d 3 and d 2; busulfan 4 mg/kg/d, d 7 to d 4. (11/9).	Chronic myeloblastic leukemia (8/8); Acute myeloblastic leukemia (3/3); Hodgkin lymphoma(6/2); Non-Hodgkin lymphoma (1/3); Acute lymphoblastic leukemia (1/0); Myelodysplastic syndrome (0/3). All patients undergoing HSCT (allogeneic or autologous).	PBM + Oral care	Oral Care	PBM was started on the first day of the conditioning (D-7) and stopped on the day of neutrophil recovery.	The patients in the laser group who had presented with erythema or ulcers continued to receive the preventive laser with 4 J/cm². A crossover was allowed for patients from the control group who presented with a grade IV OM index of the WHO and/or an ulcer area more than or equal to 12 cm according to the OMAS. A therapeutic laser with 8 J/cm2 per point was applied to these patients.	WHO, OMAS and VAS
Khouri VY et al., 2009 (Brazil) [25]	Unblinded randomized clinical trial	Mean age: ≅ 30.34	12 (2 F/10 M); 10 (3 F/7 M)	Cyclophosphamide (D-5 to D-2, 50 mg/kg): (2/0); Busulfan (D-7 to D-4, 1 mg/kg) with cyclophosphamide (D-3 to D-2, 60 mg/kg): (6/1); Busulfan (D-6 to D-3, 1 mg/kg) with fludarabine (D-6 to D-2, 30 mg/m2): (3/2); TBI (D-6 to D-4, 990 cGy total) and cyclophosphamide (D-3 to D-2, 60 mg/kg): (1/4); Fludarabine (30 mg/m2)/citarabine (2000 mg/m2)/mitoxantrone (10 mg/m2) from D-10 to D-7 and melfalan (D-3 to D-2, 70 mg/m2): (0/1). All patients were subjected to treatment with MTX in combination with cyclosporine after transplantation for prevention of GVHD.	Acute myeloid leukemia (5/3); Acute lymphoid leukemia (1/2); Chronic myeloid leukemia (3/2); Severe aplastic anemia (2/2); Myelodysplastic syndrome (1/1).	PBM + Mucositis Formula (0.15 g benzidamine, 1.13 g nistatin, 2 g neututocain and 10 mL distilled water).	Mucositis Formula (0.15 g benzidamine, 1.13 g nistatin, 2 g neotutocaine and 10 mL distilled water).	For both groups started on the first day of conditioning and lasted until the initial clinical manifestation of OM. The CG received neither laser nor placebo laser.	For both groups, it started with the initial clinical manifestations of mucositis, with monitoring until D + 15 after the transplant, and consisted of combining the standard oral hygiene protocol with the use of the “Formula for Mucositis” mouthwash. IG: Continuous laser + “Mucositis Formula”. CG: “Mucositis Formula”.	WHO Oral Toxicity Scale and OMAS
Ferreira B et al., 2015 (Brazil) [26]	A randomized, parallel, superiority trial with two arms.	> 18	17 (7 F/10 M); 18 (10 F/8 M)	Pretransplant conditioning regimes: Busulfan (1.0 mg/kg/dose) + cyclophosphamide (60 mg/kg/day) (1/3); Busulfan (1.0 mg/kg/dose) + fludarabine (150 mg/kg over 5 days (30 mg/kg/day) (6/7); BCNU (300 mg/m2), etoposide (200 mg/m2), ara-C (200 mg/m2), melphalan (BEAM) (140 mg/m2) (4/4); Melphalan (100 mg/m2/day) (5/4); Others (1/0).	Leukemia (7/7); Lymphoma (5/5); Myeloma (5/4); Others (0/2); All underwent HSCT: Autologous (12/10) and Allogeneic (5/8).	PBM + Oral care	Simulated PBM	PBM was applied from the first to the fifth day of pretransplant conditioning.	Patients in either group who went on to develop grade II OM were treated with PBM using identical parameters until the lesions had healed completely, as well as they received the same regiment protocol for pain control in which consisted of oral or subcutaneous opioids.	WHO to evaluate OM and VAS to assess pain.
Boris SP et al., 2016 (Russia) [27]	Prospective, longitudinal, controlled and randomized study	≅ 1–17	17 (8 F/9 M); 16 (6 F/10 M)	47 courses of HD intravenous methotrexate (HD-MTX)/46 courses of HD intravenous methotrexate (HD-MTX).	Acute Lymphoblastic Leukemia (6/9); T/B Lymphoma (6/3); Burkitt lymphoma (1/3); Acute Lymphoblastic Leukemia relapse (4/1).	PBM + Oral care	Oral care: descontamination and oral hygiene, intravenous administration of immunoglobulins, antimicrobial, antifungal and antiviral agents.	1 st day of the HD-MTX course before the introduction of MTX, then on the 3rd, 5th, 7th and 9th days, as long as OM did not develop, or until manifestations of its clinical signs.	The PBM procedures continued in the same way, but with a therapeutic purpose, being performed daily until the lesions healed./When severe OM occurred, patients received narcotic analgesics, enteral nutrition, and supportive anti-infective medications (Did not apply laser as therapeutic treatment).	WHO Oral Toxicity Index (OM) and SKLansky Index (Quality of life).
Elkady R et al., 2024 (Egypt) [28]	Two-arm parallel, double-blind, randomized, controlled clinical trial.	3–18	21;21 (There was no specification of sex)	Cytarabine/Mitoxantrone (All cases in the two groups)	Acute Myeloid Leukemia (All cases in the two groups)	Oral care + PBM	Oral care + Simulated PBM	Day 1 before the beginning of CT. This procedure was repeated on days 2,3,4 and 5.	In case of developing OM patients were treated as per the institution guidelines for management of OM with or without pain killers.	WHO and the scale NCI: Common Terminology Criteria for Adverse Events, version 4.0 to evaluate OM.

*IG* Intervention Group, *CG* Control Group, *LLLT* Low-Level Laser Therapy, *CT* Chemotherapy, *OM* Oral Mucositis, *WHO* World Health Organization, *NCI* National Cancer Institute, *F* Female, *M* Male, *HSCT* Hematopoietic stem-cell transplantation, *VAS* Visual Analog Scale, *COMS* Cumulative Oral Mucositis Score, *MTX* Methotrexate, *GVHD* Graft-Versus-Host Disease, *OMAS* Oral Mucositis Assessment Scale, *TBI* Total Body Irradiation, *HD* High-Dose, *DMI* Daily Mucositis Index, *BMT* Bone Marrow Transplant, *PBM* Photobiomodulation

Cowen et al. [23] followed 30 patients aged 18–58 years, in two groups (15 in the PBM group and 15 in the control/simulated PBM group), diagnosed with acute lymphoblastic leukemia, acute myeloblastic leukemia, non-Hodgkin’s lymphoma and multiple myeloma. All individuals underwent HSCT and were previously conditioned with: intravenous cyclophosphamide (D-5, D-4) or intravenous melphalan (d-5). The He-Ne laser group received application for five consecutive days (from day − 5 to day − 1; before transplantation), in five anatomical sites on the right and left side of the oral cavity: lower labial mucosa and adjacent gum, upper labial mucosa and adjacent gum, buccal mucosa and adjacent gum, ventral and lateral tongue and floor of the mouth (15 points in each anatomical site), with a wavelength of 632.8 nm, 10 s per point and 54 J of total energy. The control group was treated with a sham laser. Therefore, at the end of 21 days, a cumulative OM score was created, demonstrating the effect of PBM in reducing the maximum intensity of OM. Furthermore, daily cumulative pain scores, swallowing ability, and saliva production were better in the intervention group than in the control group.

Antunes et al. [24] analyzed the clinical effects of PBM in preventing and reducing OM induced by conditioning for HSCT. For this purpose, 38 patients were randomized into two groups (19 in the PBM group and 19 in the placebo group), with an average age of 36.5 years. Presenting diagnoses of hematologic cancers or hematologic diseases (Chronic or Acute Myeloblastic Leukemia; Hodgkin’s or non-Hodgkin’s Lymphoma; Acute Lymphoblastic Leukemia; and Myelodysplastic Syndrome). In the PBM group, the 660 nm InGaAlP laser was applied to 15 points per region for 16.7 s on the upper lip, lower lip (redness and lip mucosa), buccal mucosa, dorsum, ventral and lateral tongue, the floor of the mouth, and the hard and soft palates. Application began on day 1 of conditioning (D-7) and was discontinued on the day of neutrophil recovery. The placebo group did not receive PBM. The patients in the laser group who had presented with erythema or ulcers continued to receive the preventive laser with 4 J/cm². A crossover was allowed for patients from the control group who presented with a grade IV oral mucositis index of the World Health Organization (WHO) and/or an ulcer area more than or equal to 12 cm according to the Oral Mucositis Assessment Scale (OMAS). A therapeutic laser with 8 J/cm² per point was applied to these patients. In summary, the results of the study showed that the use of PBM had potential in reducing the incidence and severity of OM.

Khouri et al. [25] conducted a study with 22 patients undergoing allogeneic HSCT, diagnosed with hematologic cancer or hematologic diseases (Acute Myeloid Leukemia, Acute Lymphocytic Leukemia, Chronic Myeloid Leukemia, Severe Aplastic Anemia and Myelodysplastic Syndrome) to compare the frequency and evolution of OM, dividing the participants into two groups. The group undergoing PBM (*n* = 12) and the group undergoing only conventional therapy with “mucositis formula” (*n* = 10) underwent a conditioning regimen with High Doses (HD) of CT (Cyclophosphamide, Busulfan and Fludarabine), with different combinations. All patients underwent conditioning regimens with cyclophosphamide associated with other CT agents, and underwent treatment with Methotrexate (MTX) in combination with cyclosporine after HSCT. In the PBM group, InGaAlP lasers (660 nm - red laser) and GaAlAs lasers (780 nm - infrared laser) were applied for 10 s at each point, the application was from the beginning of the conditioning regimen until D + 15 post-transplant, on alternate days, in the following locations: upper and lower lips (vermilion and mucosa), bilateral buccal mucosa, floor of the mouth, lateral surface of the tongue on both sides and ventral surface of the tongue. In the conventional therapy group, the “mucositis formula” consisted of 0.15 g of benzydamine, 1.13 g of nystatin, 2 g of neotutocaine, and 10 mL of distilled water. According to the present study, the PBM group showed a lower frequency and progression of OM, as well as a reduction in the number and severity of lesions, when compared to the conventional therapy group.

Ferreira et al. [26] conducted an RCT with 35 patients (17 in the PBM group and 18 in the placebo group) with hematologic cancer (Leukemia, Lymphoma, Myeloma, and others), with a mean age of 42.44 years, undergoing CT protocols prior to autologous and allogeneic HSCT. The protocols used were Busulfan + Cyclophosphamide, Busulfan + Fludarabine, Carmustine (BCNU), Etoposide, Ara-C, Melphalan (BEAM); Melphalan and others. In the PBM group, applications were obtained at 27 points in the oral cavity, using the InGaAlP laser, with a wavelength of 650 nm, for 20 s per point. Applications were made to the upper and lower labial mucosa; bilateral buccal mucosa; dorsal, ventral and lateral surfaces of the tongue; retromolar region; soft palate; and floor of the mouth. The application was carried out from the 1 st to the 5th day of pre-transplant conditioning. In the placebo group, sham treatments were performed, with the laser on but no light emission. As a result, no statistically significant difference in the incidence of OM was observed between the groups, but PBM contributed to a reduction in the progression to severe OM and a decrease in pain intensity.

Boris et al. [27] included 33 pediatric patients, aged 1 to 17 years, in two groups (17 in the laser group with 47 courses of HD-MTX and 16 in the comparison group with 46 courses of HD-MTX), diagnosed with Acute Lymphoblastic Leukemia and non-Hodgkin’s Lymphoma.Patients were treated according to the following protocols: ALL-MB-2008 and ALL-BFM-2002-Rez, B-NHL-M-2010, NHL-BFM-95, including courses (two to four) of HD-MTX (1, 2, or 5 g/m2). The intervention group received preventive PBM on the 1 st day of the HD-MTX course (before the CT infusion), and afterwards, on the 3rd, 5th, 7th and 9th day. If OM developed, the protocols were performed therapeutically. Thus, the 670 nm wavelength was used in 13 zones of the oral mucosa susceptible to OM was irradiated sequentially: on the left and right - the tissues of the cheek along the line of occlusion of the teeth, the retromolar space, the lateral and ventral surfaces of the tongue, as well as the palate, upper and lower lips for 12 s per point. For therapeutic purposes, the laser was applied directly to the mucositis foci, with the lesion area divided into multiple sub-areas of 0.5 cm², each point being irradiated for 12 to 24 s in confirmed cases of OM. The control group received only oral care. In summary, PBM reduced the incidence of OM, especially during HD-MTX use. However, despite alleviating the progression of inflammation, it did not show a statistically significant difference in the frequency of severe OM or in the reduction of oral cavity recovery time.

Elkady et al. [28] evaluated 42 patients (21 from the PBM group and 21 from the control group), aged between 3 and 18 years, diagnosed with Acute Myeloid Leukemia and treated with protocols containing Cytarabine and Mitoxantrone. In the PBM group, 44 points in the oral cavity: 8 points in the right buccal mucosa, 8 in the left buccal mucosa, 4 in the upper labial mucosa, 4 in the lower labial mucosa, 6 in the dorsum of the tongue, 6 in the floor of the mouth and 4 in the soft palate with a wavelength of 660 nm, for 10 s per point. These procedures were performed on days 1, 2, 3, 4 and 5, with the assessment of OM degrees carried out on the 5th, 12th, 19th and 30th day. In the control group, oral care and simulated PBM were performed, with analyses being performed on the same days mentioned above. According to the trial, the laser group presented a lower incidence of mucositis in grades I, II and III, on the 12th and 19th day. Furthermore, PBM was considered effective, safe and viable for preventing OM in children treated with CT.

Heterogeneity in the methods used to assess outcomes among the included studies was observed. Cowen et al. [23] used a pre-established scale, as well as the Daily Mucositis Index (DMI) and Cumulative Oral Mucositis Score (COMS). Antunes et al. [24] used the WHO scale, the OMAS, and the Visual Analog Scale (VAS). Similarly, Khouri et al. [25] applied the WHO scale and the OMAS, while Ferreira et al. [26] chose the WHO to assess OM and the VAS to analyze pain. Boris et al. [27] used the WHO Oral Toxicity Index associated with the Sklansky Index to assess QOL. Finally, Elkady et al. [28] used the WHO scale together with the Common Terminology Criteria for Adverse Events (NCI-CTCAE, version 4.0) to measure OM.

In view of the above, it is observed that the red wavelength was used in all RCTs, ranging from 632.5 nm to 670 nm. Furthermore, only the study by Khouri et al. [25] associated the red and infrared wavelengths (780 nm) on alternate days. For other dosimetric parameters of PBM and number of application points/zones in the oral cavity, there was no agreement between the protocols mentioned in Table 3. Regarding the outcomes of the selected studies, PBM proved to be effective in reducing the incidence and/or frequency, in addition to preventing the progression to more severe cases of OM (Table 4).

**Table 3:** Laser therapy parameters of the studies

Author/year	Laser type	Spot size cm2	Energy per point (J)	Energy per session (J)	Power (mW)	Dose (J/cm2)	Λ (nm)	Mode of application	Time of exposure per point (s)
Cowen D et al.., 1997 (France) [23]	Helium-neon (He-Ne) laser	NR	0.6	54	60	1.5	632.8	Intraoral and continuous emission.	10
Antunes HS et al., 2007 (Brazil) [24]	InGaAlP diode laser	0.196	0.8	11.5	46.7	Preventive: 4 (Laser group) Treatment: 8 (Control group)	660	Intraoral and continuous emission.	16.7
Khouri VY et al., 2009 (Brazil) [25]	Twin Laser: InGaAIP and GaAlAs	NR	0.25	≈ 2.0	25	6.3	660 nm (InGaAlP - red laser) and 780 nm (GaAlAs - infrared laser)	Intraoral, with direct contact with the mucosa. The two lasers were applied on alternate days and in the morning.	10
Ferreira B et al., 2015 (Brazil) [26]	InGaAlP laser - Therapy XT-DMC	0.028	2	54	100	70	650	Intraoral, continuous, applications were precise, perpendicular and in direct contact with the irradiated area.	20
Boris SP et al., 2016 (Russia) [27]	Semiconductor diode laser - “Снаг-Сенс-К” (Luzar, Belarus)	0.5	Preventive and therapeutic (intraoral): 0.72 Therapeutic (transcutaneous): 1.44.	Preventive: ≈ 33; Therapeutic: ≈ 28 to 50, depending on the number of lesions and the method of application.	30	Preventive: 5.16; Therapeutic: 5.16–21.24, varied according to the number of injured areas and the number of applications accumulated in the treatment sessions.	670	Intraoral, in continuous mode, however, in cases of severe trismus, the procedure was performed transcutaneously for double the duration.	Preventive and therapeutic (intraoral): 12 Therapeutic (transcutaneous): 24
Elkady R et al., 2024 (Egypt) [28]	Diode laser (Sirrolaser Blue™, USA)	0.08	0.25	11	25	3.1	660	Intraorally, perpendicularly, continuously, with the tip of the device covered with translucent plastic and applied to the oral cavity, previously dried with gauze.	10

*NR* Not reported

**Table 4:** Outcome and conclusion of studies

Author/year	Main outcome (Prevention of OM)	Secondary outcome	Conclusions
Cowen D et al.., 1997 (France) [23]	Laser application significantly reduced the intensity and severity of OM, with a lower mean score on the COMS scale (132.3 ± 21.9 vs. 225.7 ± 40.5; *p* = 0.04), and fewer days with grade III OM (0.69 ± 1.4 vs. 2.41 ± 2.3; *p* = 0.01).	Laser applications diminished pain: mean severity of pain was 12.7 ± 1.3 for laser group patients versus 20.3 ± 2.5 for placebo group patients (*p* = 0.05).	Prophylactic use of low-level helium-neon (He-Ne) laser was well tolerated, safe, feasible in all cases, and effective in reducing HD-CT-induced OM before autologous BMT.
Antunes HS et al., 2007 (Brazil) [24]	Patients who underwent PBM showed lower intensity of OM (grades 0–I; *p* < 0.001) and a lower risk of developing grade II–IV mucositis (HR = 0.41; *p* = 0.002), with an even more significant reduction for grades 3–4 (HR = 0.07).	The control group showed a longer healing time, with no statistically significant difference, and there was no difference in the presence or intensity of pain between the groups, with pain reported in 14 (73.7%) patients in the laser group (VAS 7) and 16 (84.2%) in the control group (VAS 8) (*p* = 0.13).	The results indicate that the initial use of PBM in patients undergoing HSCT is a powerful tool in reducing the incidence of OM.
Khouri VY et al., 2009 (Brazil) [25]	The PBM group presented lower frequency and lower severity of oral mucositis compared to the control group, with statistically significant differences in frequency (*p* = 0.02), mean grade on the WHO scale (1.75 ± 0.45 vs. 2.45 ± 0.93; *p* < 0.01) and score on the OMAS scale (7.0 ± 3.2 vs. 14.0 ± 8.3; *p* = 0.01).	Regarding the answers to the questionnaire applied in the PBM group, 50% of the patients reported fewer lesions, 10% more lesions, 75% reported not requiring analgesics for oral pain, 25% reported use the analgesics to oral pain, 100% pain to swallowing, 100% improvement after laser applications.	PBM reduced the frequency, progression, and severity of OM compared to conventional therapy with the “*Mucositis Formula*”, indicating its potential as a standard method for prevention and treatment of the condition after allogeneic HSCT.
Ferreira B et al., 2015 (Brazil) [26]	The incidence of grade II OM was 71.43%, with no difference between the groups (*p* = 0.146), however, severe OM (grades 3–4) was significantly lower in the PBM group (17.65%) compared to the control group (61.11%) (*p* = 0.015). Furthermore, PBM increased lesion-free survival, with the development of severe OM occurring in less than 40% of cases in the intervention group versus 100% in the control group (*p* = 0.0397).	Treatment of OM with PBM, initiated after the appearance of lesions, was effective in promoting complete healing, reducing progression to severe OM and decreasing pain intensity in patients in both groups.	In conclusion, these results appear to favor the implementation of PBM as a safe and effective alternative for the prevention of severe OM and pain in patients submitted to HSCT.
Boris SP et al., 2016 (Russia) [27]	The use of PBM led to a statistically significant decrease in the incidence of OM during CT courses (from 67.4 to 30%; *p* < 0.001). The use of PBM reduced the risk of OM during a course of HD-MTX CT by 88% (95% CI 40–98; *p* < 0.001).	PBM significantly reduced pain (VAS: 0.8; 95% CI: 0–1.8 vs. 2.3; 95% CI: 1.2–3.4; *p* = 0.021) and improved quality of life (Lansky Index: 91; 95% CI: 94–99 vs. 63; 95% CI: 54–71; *p* < 0.001), with good acceptance and safety, without significantly reducing the duration of OM or impacting overall survival.	The systematic use of PBM was accompanied by a reduction in the frequency of OM, a decrease in pain intensity and an increase in the QOL of children, as well as significant cost savings.
Elkady R et al., 2024 (Egypt) [28]	The laser-treated group showed a higher prevalence of patients without OM compared to the placebo group on days 12 (*p* < 0.001; effect size = 0.796), 19 (*p* < 0.001; effect size = 0.670), and 30 (*p* < 0.001; effect size = 0.576), while the placebo group showed a higher prevalence of cases with OM in grades I–III throughout the follow-up period.	NR	Finally, it was concluded that PBM is safe, feasible, and effective and should be introduced as a standard prevention approach for CT-induced OM in pediatric patients.

*NR* Not reported, *WHO* World Health Organization, *OMAS* Oral Mucositis Assessment Scale, *OM* Oral Mucositis, *MTX* Methotrexate, *HD* High-Dose, *CT* Chemotherapy, *HSCT* Hematopoietic stem-cell transplantation, *VAS* Visual Analog Scale, *COMS* Cumulative Oral Mucositis Score, *HD* High-Dose, *BMT* Bone Marrow Transplant, *HR* Hazard Ratio, *QOL* Quality Of Life, *PBM* Photobiomodulation, *CI* Confidence interval

### Assessment of risk of bias

Overall, the included RCTs demonstrated a predominance of low risk of bias across most evaluated domains. Among the six studies analyzed, the studies by Cowen et al. [23], Antunes et al. [24], Ferreira et al. [26] and Elkady et al. [28] achieved low risk of bias in most domains, including the “Outcome Measurement” domain, in which the evaluators who performed the follow-up and outcome assessments were blinded to treatment allocation. This level of rigor minimized the potential for detection bias, increasing the reliability of their findings.

The studies conducted by Boris et al. [27] and Khouri et al. [25] presented a high risk of bias, especially in the Randomization process and Measurement of outcome domains. This risk stems largely from the lack of blinding in the outcome assessment, which can introduce subjective influence in the interpretation of results. Prior knowledge about the intervention received by participants can affect how measurements are recorded or interpreted, compromising the accuracy and impartiality of the findings and, consequently, the methodological robustness of the study and the reliability of the data obtained.

All studies presented some concerns about bias, specifically in the “Selection of the reported outcome” domain, likely due to the lack of a pre-specified analysis plan prior to data collection or analysis and the performance of multiple analyses/outcomes. Despite this limitation, the studies presented a low risk of bias in the “Deviations from the intended interventions” and “Missing outcome data” domains, thus strengthening the internal validity of the study and the credibility of the conclusions.

**Fig. 2:**
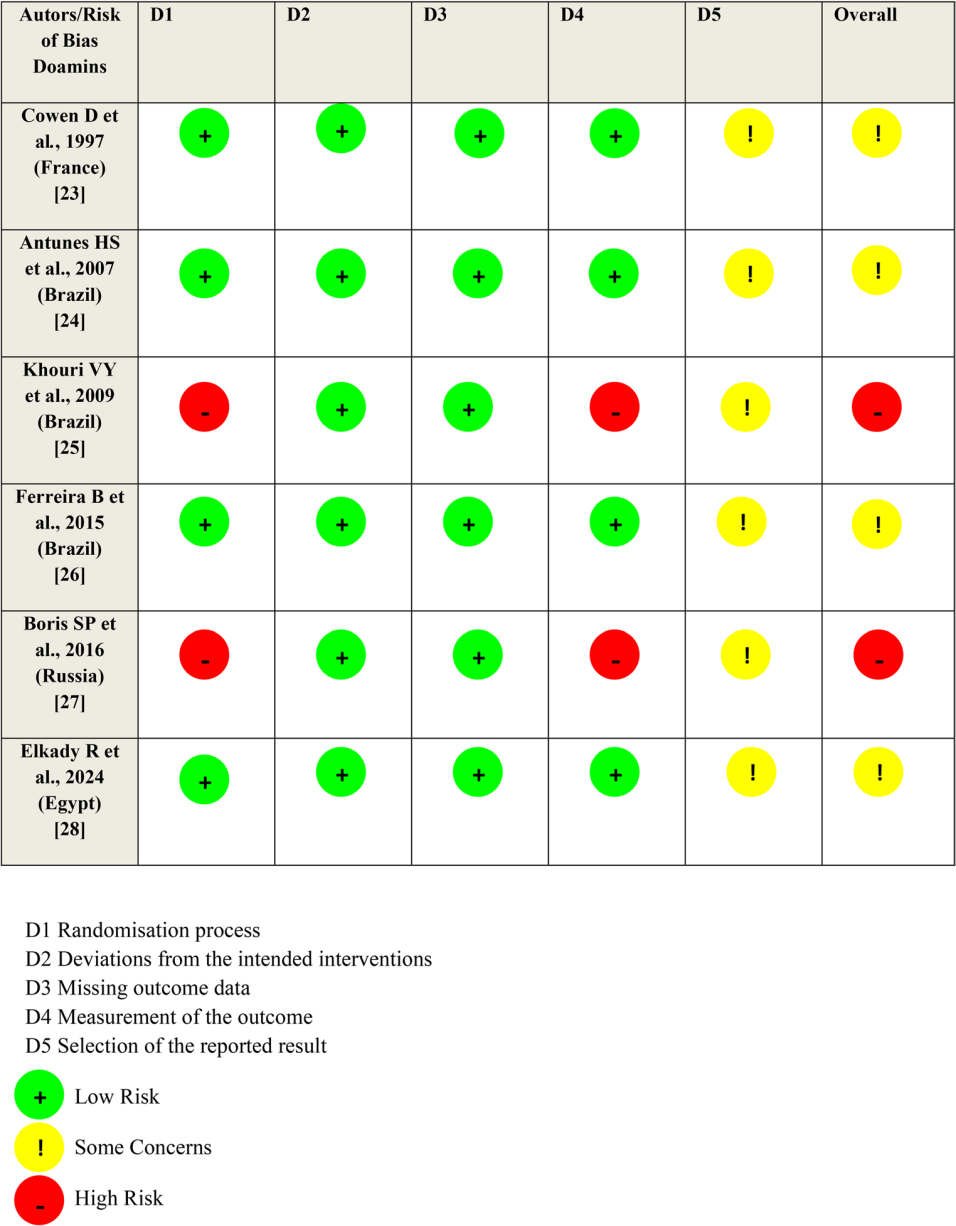
Assessment of risk of bias based on Cochrane risk of bias tool for randomized trials (RoB 2.0)

In summary, although the overall methodological quality of the included RCTs was strong, with a low risk of bias in critical areas such as randomization and data completeness, the lack of blinding in outcome assessment and selection of reported results was a limitation of some included studies. This highlights the need for future studies to further prioritize blinding procedures to increase the reliability and validity of their findings, as well as to adopt prespecified analysis plans, record all outcomes and measures transparently, and fully report all planned results, avoiding selective selection of outcomes and analyses based on the obtained findings (Fig. 2).

### Certainty of evidence

Table 5 summarizes the quality of the evidence. The certainty of the evidence, based on the GRADE approach, indicated moderate certainty for the prevention of OM with PBM in the included RCTs.The evidence was downgraded for imprecision, but was not inconsistent or indirectly relevant to the broad question. A large magnitude effect was also observed, which contributed to increased confidence in the findings. This means that, although there are methodological limitations and undersized samples, the studies show consistent and clinically relevant results, which can be applicable to the population, intervention and outcomes of interest in clinical practice.

**Table 5:** Evidence certainty assessment with grading of recommendations, assessment, development, and evaluation (GRADE)

Certainty Assesment – Prevention of oral mucositis							
**No Of Studies**	**Study Design**	**Risk of Bias**	**Inconsistency**	**Indirectness**	**Imprecision**	**Other Considerations**	**Certainty**
6	RCT	Not serious^a^	Not serious^b^	Not serious^c^	Very serious^d^	Strong association^e^	⊕⊕⊕Ο Moderate

(a) Most of the included studies raised some concerns overall

(b) There was no great variation between the results of the studies

(c) The assessment of indirectness considered the population, intervention, comparator and outcome between studies, without restricting specific variables

(d) Studies with small samples; significant results, but with undersized samples

(e) Publications bias undetected, large effect, no plausible confusion and no dose response gradient

## Discussion

In order to evaluate the preventive efficacy of PBM in OM in patients with hematologic diseases undergoing CT, the present research sought to include RCTs that mainly relied on the preventive perspective of this therapy. Although there are already reviews with a similar theme, such as that by Franco et al. [31], which exclusively analyzed PBM for the treatment of HSCT-induced OM, and that by He et al. [32], which evaluated the effect of PBM on CT-induced OM only in pediatric and young patients, based on the available evidence, this is the first systematic review that brings together, from RCTs, evidence on the preventive use of PBM in individuals with hematologic diseases (benign or malignant), of any age group, and regardless of HSCT.

For the diagnosis of OM, most studies took into account the WHO Oral Toxicity Index criteria. In addition to these criteria, Antunes et al. [24] and Khouri et al. [25] also used the OM assessment using the OMAS criteria, and Elkady et al. [28] took into account the NCI scale. In agreement with the results obtained in other systematic reviews [31–33] and corroborating the MASCC/ISOO guidelines [34] the present study showed that prophylactic PBM was effective in reducing the incidence and/or frequency of OM in hematologic patients undergoing CT and preparing for HSCT. Furthermore, it is suggested that such results were also influenced by the maintenance of oral hygiene, since all patients in the studies analyzed were instructed on oral care. Reinforcing the findings of the literature, which emphasizes the role of PBM in tissue repair, reduction of severity and decrease of OM pain [35–37], and shows the maintenance of good oral hygiene as an agent of OM prevention [38]. In contrast, the studies by Cruz et al. [39] and Simões et al. [40] did not find any benefits from the application of PBM to reduce the severity of OM, and the results by Amadori et al. [41] were not sufficient to conclude the benefit of PBM against OM.

The increase in pro-inflammatory cytokines in hematologic patients with neutropenia was considered a risk factor for OM [42]. Since such blood alteration compromises the protective capacity of the oral mucosa and increases the risk of microbial colonization. Meanwhile, Curra et al. [43] evaluated the hematological toxicity associated with the severity of OM, it was observed that patients with lower levels of leukocytes, platelets and hemoglobin presented severe OM (grade III and IV), while for each increased unit of leukocytes, the severity of OM was reduced. Fidan and Arslan [42] considered in their studies the presence of leukopenia as a risk factor for OM. However, of the articles evaluated in our study, only Antunes et al. [24] cited the analysis of neutrophils in response to OM, without associating neutrophil recovery with rapid OM resolution. Thus, there is a need for further studies to better understand the relationship between neutrophil recovery and OM healing. Regarding the mechanism of PBM in cells, He et al. [32] observed some inconsistencies, which are not well understood.

Another factor to be considered is the association between the degree of OM and the chemotherapy protocol, in which, in the studies carried out by Curra et al. [43], patients who used combined protocols with MTX-HD, doxorubicin and cyclophosphamide; and MTX-HD and cyclophosphamide presented more severe degrees of OM. In the present review, in the studies by Khouri et al. [25] and Boris et al. [27], all patients evaluated underwent treatment with MTX, presenting an increased risk for OM. Furthermore, since OM is a common complication in patients undergoing HSCT, the incidence of OM is also influenced by the dosage of the chemotherapy agents used [44]. Furthermore, allogeneic HSCT is considered an increased risk factor for the severity of OM when compared with autologous HSCT [24, 42]. Among the articles evaluated, only that by Khouri et al. [25] shows that all participants underwent allogeneic HSCT, belonging to the highest risk group.

Patient age is a relevant risk factor for the development of OM, since younger individuals have greater mitotic activity of the oral mucosa, which increases susceptibility to the cytotoxic effects of chemotherapy, while also favoring faster tissue repair [45, 46]. In the studies by Hespanhol et al. [46], a higher incidence of OM was observed in pediatric patients compared to adolescents and adults undergoing similar therapeutic protocols, with greater involvement among patients aged 0 to 10 years, representing 27% of cases, compared to those aged 11 to 20 years, who represented 18% of the patients evaluated. In this context, when evaluating the effects of PBM on oral mucositis, the present systematic review included patients of all age groups, allowing for a comprehensive analysis independent of age. However, potential age-related variations in tissue response and therapeutic parameters reinforce the need for protocol standardization and clinical trials that directly compare outcomes across different age groups.

In the protocols used in laser applications, all RCTs used the red wavelength, with variations between 632.8 and 670 nm, except for one [25] which associated the red and infrared wavelengths (780 nm). The irradiation time per point varied between 10 and 20 s, as the minimum and maximum time, respectively. Energy parameters per session and power were the ones that presented the most discrepancies, with differences of up to 75 mW between the studies by Ferreira et al. [26, 28]. Irradiation occurred intraorally in all selected studies, but the number of points/zones was not the same between the prophylactic protocols. In addition, the frequency of application also did not follow the same pattern, probably due to the difference between the CT regimens employed. According to Bjordal et al. [47], the ideal time to start laser therapy sessions with a focus on preventing OM is 7 days before CT regimens. However, in this review, the time varied between D-7, D-5 in pre-HSCT conditioning and D-1 before CT infusion. This information suggests the need for standardization of laser therapy parameters for the prevention of OM. According to He et al. (2018), a dose greater than 2 J per point, for more than 10 s, is recommended for prophylactic purposes. However, exaggerated stimuli can influence cell viability and, consequently, the prevention of tissue damage, since there is a fine line between the biostimulation capacity and biosuppression of PBM [29].

Some authors have used sham laser therapy, with or without oral care, as a control group management. In Cowen et al. [23], the sham application reproduced noises, probe positioning, and the use of protective eyewear, without therapeutic emission, ensuring blinding of patients and evaluators. Ferreira et al. [26] used a laser switched on without therapeutic light emission, with automatic shutdown, simulating application to the oral mucosa. Elkady et al. [28] employed equipment switched on only with the visible targeting beam, without laser activation. These results in these studies corroborated the high incidence, severity, and pain scores associated with the group undergoing sham laser therapy, as well as the longer lesion-free survival time in the intervention group. These data reinforce that the group treated with laser obtained superior results in the prophylactic intervention for oral mucositis when compared to the groups undergoing sham laser therapy or without laser [23, 26, 28].

QOL was a subjective criterion used by the studies. Boris et al. [27] cited the SBLansky index scale and the VAS as an evaluation method, according to the authors the reduction in pain intensity resulted in a greater QOL for the PBM group. The same occurred with Antunes et al. [24] and Ferreira et al. [26] when they stated that pain intensity was lower with the use of PBM, however the use of morphine can be considered a confounding bias for data analysis. The study by Antunes et al. [24] deserves highlighting, as they applied a higher effective dose (8 J/cm²) with therapeutic intent in patients in the control group, initiating PBM only after the development of grade IV OM or an ulcerated area ≥ 12 cm². This approach, clearly focused on treatment and not prevention, proved effective, with clinical recovery in about six days, suggesting a benefit of using higher doses in the management of severe OM cases. Therefore, it is proposed that factors such as the therapeutic intent of PBM, the dosimetry employed, and the concomitant use of narcotics exert a significant influence on both the clinical response and the subjective perception of the benefits of PBM, including improved QOL [30].

Some limitations were observed during the analysis of the selected articles. The lack of standardization of chemotherapy regimens and laser therapy protocols, small sample sizes, and a small number of studies, in addition to aspects inherent to the risk of bias, such as lack of blinding and selection of reported results, reflect a moderate certainty of evidence, which requires caution and critical interpretation of the results presented. This data may suggest and influence future changes for future research on the topic.

## Conclusion

Through analysis and interpretation of the data obtained, PBM appears to be effective in preventing and reducing the frequency of OM in patients with hematologic diseases undergoing CT, reducing the incidence and severity of the condition. Furthermore, pain, quality of life, and the therapeutic use of PBM also demonstrated clinical benefits. The variability in laser application protocols reinforces the need for standardized RCTs in order to consolidate evidence and guide clinical practice. 

## Data Availability

No datasets were generated or analysed during the current study.
